# A dendritic/tumor fusion cell vaccine enhances efficacy of nanobody-based CAR-T cells against solid tumor

**DOI:** 10.7150/thno.84946

**Published:** 2023-09-18

**Authors:** Shuyang Sun, Ziqiang Ding, Li Gao, Bruce D. Hammock, Xianing Huang, Zhi Ping Xu, Xuan Wang, Qihong Cheng, Fengzhen Mo, Wei Shi, Shenxia Xie, Aiqun Liu, Haixia Li, Xiaomei Yang, Xiaoling Lu

**Affiliations:** 1 College of Stomatology/ Hospital of Stomatology/ School of Basic Medical Sciences/ Guangxi Key Laboratory of Nanobody Research/ Guangxi Nanobody Engineering Research Center/ Laboratory Animal Center/ Pharmaceutical College/ Affiliated Tumor Hospital, Guangxi Medical University, Nanning, 530021, China.; 2 UCD Comprehensive Cancer Center, Department of Entomology and Nematology, University of California, Davis, CA 95616, USA.; 3 Australian Institute for Bioengineering and Nanotechnology, University of Queensland, Brisbane, QLD 4072, Australia.; 4 Department of Neurosurgery, Union Hospital, Tongji Medical College, Huazhong University of Science and Technology, Wuhan, 430022, China.

**Keywords:** nanobody, CAR-T cells, DC/tumor fusion vaccines, EGFRvIII, solid tumor

## Abstract

**Background:** Chimeric antigen receptor (CAR) T-cell therapy is practical in treating cancers of hematopoietic origin, but of that in solid tumors compromises efficacy for the loss of the antigen recognized by the CAR. However, dendritic cell (DC)/tumor fusion vaccines present a spectrum of known or unknown tumor antigens to stimulate T cell expansion and enhanced T cell response. Developing a new strategy of enhanced nanobody-based CAR-T (Nb-CAR-T) cells antitumor activity by DC/tumor fusion vaccines stimulation would provide guidance for more effective CAR-T cell therapies.

**Methods:** Considering the therapeutic potential of nanobody (Nb), we first screened EGFRvIII Nb, then constructed and verified the function of EGFRvIII Nb-CAR-T cells *in vitro* and *in vivo*. We further combined DC/tumor fusion vaccines to boost EGFRvIII Nb-CAR-T cells antitumor effect, which was evaluated* in vitro* Nb-CAR-T cell function and in the tumor-bearing xenograft mouse models.

**Results:** We had for the first time successfully selected EGFRvIII Nb for the generation of the novel EGFRvIII Nb-CAR-T cells. Importantly, our results suggested that DC/tumor fusion vaccines stimulate Nb-CAR-T cells response not only in improving T cell proliferation, T cell activation, cytokine secretion and tumor-specific cytotoxicity *in vitro*, but also significantly reducing tumor burden, prolonging survival and improving Nb-CAR-T cells infiltration.

**Conclusions:** We have innovatively shown that DC/tumor fusion vaccines significantly enhance the efficacy of Nb-CAR-T cells against solid tumors. This new strategy has provided a promising therapeutic platform for promoting the clinical treatment of CAR-T cells therapy.

## Introduction

The historical outcomes for millions of patients with advanced or recurrent cancers are performed wretchedly due to resistance to conventional treatment regimens and low-rate cure 1,2. Recent years, chimeric antigen receptor (CAR) T-cell has been designed to recognize and eliminate tumor cells via bridging between tumor-associated antigens (TAAs) and has become a sound momentum of therapeutic potential in hematological malignancies clinical practice, particularly CD19 CAR-T cells therapy 3-7. However, several CAR-T cells have been described in treatment of late-stage multifocal, bulky solid tumors in recent clinical studies 8,9, the responses are not consistent and less favorable, mainly for specific and homogeneously expressed targets, and the existing strong heterogeneity of complex components within solid tumor 10,11, Therefore, new and active CAR-T cells with reliable safety and significant improvement in solid tumor treatments are urgently needed.

Apart from optimizing and modifying the structure of CARs according to solid tumors, selecting targets with adequate specificity is critical for developing effective CAR-T cell immunotherapies. The epidermal growth factor receptor variant III (EGFRvIII) has been tested successfully for CAR-T therapies against solid tumors, which is not only a cell surface oncogenic epitope expressed on 30% of glioblastomas (GBMs), but also a broad array of neoplasms existed in other tissues, including the breast, lung, liver, prostate and so on, but it is not found in any normal tissues 12,13. Exons 2-7 of EGFRvIII are deleted in frame and at the terminus of exons 1 and 8 a glycine residue is generated; such mutation is associated with tumorigenicity and affects tumor cells by conferring resistance to radiotherapy and chemotherapy 14. In GBM patients who survive more than 1 year have been diagnosed with EGFRvIII expression as being an indicator of poor prognosis 15. Based on preclinical and clinical studies, GBM treatment options especially EGFRvIII CAR-T cells are currently in progress 16,17. However, several recent phase I clinical trials of EGFRvIII CAR-T cells in patients with recurrent GBM suggested that the antitumor effect is far less than expected in early clinical studies 18,19. It is for these reasons that EGFRvIII is an ideal target for immunotherapy against cancer, and logically we have selected EGFRvIII as the candidate neoepitope for designing a target-specific recognition domain of high and specific affinity, which will enhance the antitumor function of CAR-T cells.

Nanobodies (Nbs, also referred to as VHH) are derived from camelid heavy-chain antibodies without light chains 20, which are proverbially explored for diagnostic and therapeutic agents. Nanobodies are small (~15 kDa) that retain excellent binding affinities 21,22. Typically, antigen recognition is usually carried out by single-chain variable fragments (scFvs) on CAR-T cells, which are usually presented to shuffle the heavy-light chains when making function as inaccurate misfolding and unstable aggregation resulting in limited CAR-T targeting ability. In contrast, Nbs are easy to engineer and perform as appropriate antigen recognition domains in CAR-T cells with no need for additional folding and assembly steps during recognition 23-25. Recent studies have recently shown the potential of Nb-based CAR-T cells development 26,27, they also found that Nb-based CAR-T cells had a greater transduction efficiency and more obvious tumor regression compared to scFv-based CAR-T cells in pancreatic cancer models. Therefore, the natural advantages of nanobodies may greatly improve the function of Nb-based CAR-T cells against solid tumors.

The latest strategies of tumor immunotherapy tend to utilize reinforced antigen presentation and adequate epitope spreading for engaging adaptive immune recognition and improving tumor cell killing. Apart from introducing Nb into structure optimization, we still have work to enhance CAR-T cells efficiency for resolving certain limitations with single epitope antigen recognition by Nb-CAR construct, especially in complex solid tumors. DCs serve as the most potent professional antigen presenting cells (APCs), which are the most critical factor to kill tumor cells by presenting TAAs and mediating the activation and differentiation of cytotoxic T lymphocytes (CTL) to enhance their antitumor effect 28-30. Further studies have shown that the autologous lysate vaccine (DCVax®-L) had a good effect on GBM patients 31,32. Vaccines that utilize tumor cells as the source of antigen such as a DC/tumor fusion cell (FC) vaccine could produce strong and durable antitumor immune responses for co-stimulation with antigens. Moreover, the DC/tumor fusion vaccines previously constructed by our group induced CTL-specific killing of tumor cells and demonstrated a good antitumor effect in the tumor-bearing mouse model 33-36. To enhance engraftment and persistence of Nb-CAR-T cells, especially in such a major barrier in the treatment of solid tumors, our group has first hypothesized that a conjugation of Nb-CAR-T cells with DC/tumor fusion vaccines enhances therapeutic activity in solid tumor treatments.

In this study, we aim to develop Nb-CAR-T cells to bind EGFRvIII-specific epitope expressed on GBM cells, fuse human monocyte-derived DCs with tumor cells as FCs, and finally combine Nb-CAR-T cells and FCs to efficiently treat GBM in the preclinical mouse model. Our results have proved our hypothesis that the combination therapy significantly enhanced the therapeutic efficacy because this strategy bundles and superposes the advantages of Nb-CAR-T cells therapy and DC/tumor fusion vaccines. Therefore, our strategy of enhancing Nb-CAR-T cells induced by DC/tumor fusion vaccines will provide a potential therapeutic platform against solid tumor immunotherapy.

## Results

### Library construction and functional verification of EGFRvIII Nb

**Figure 1:**
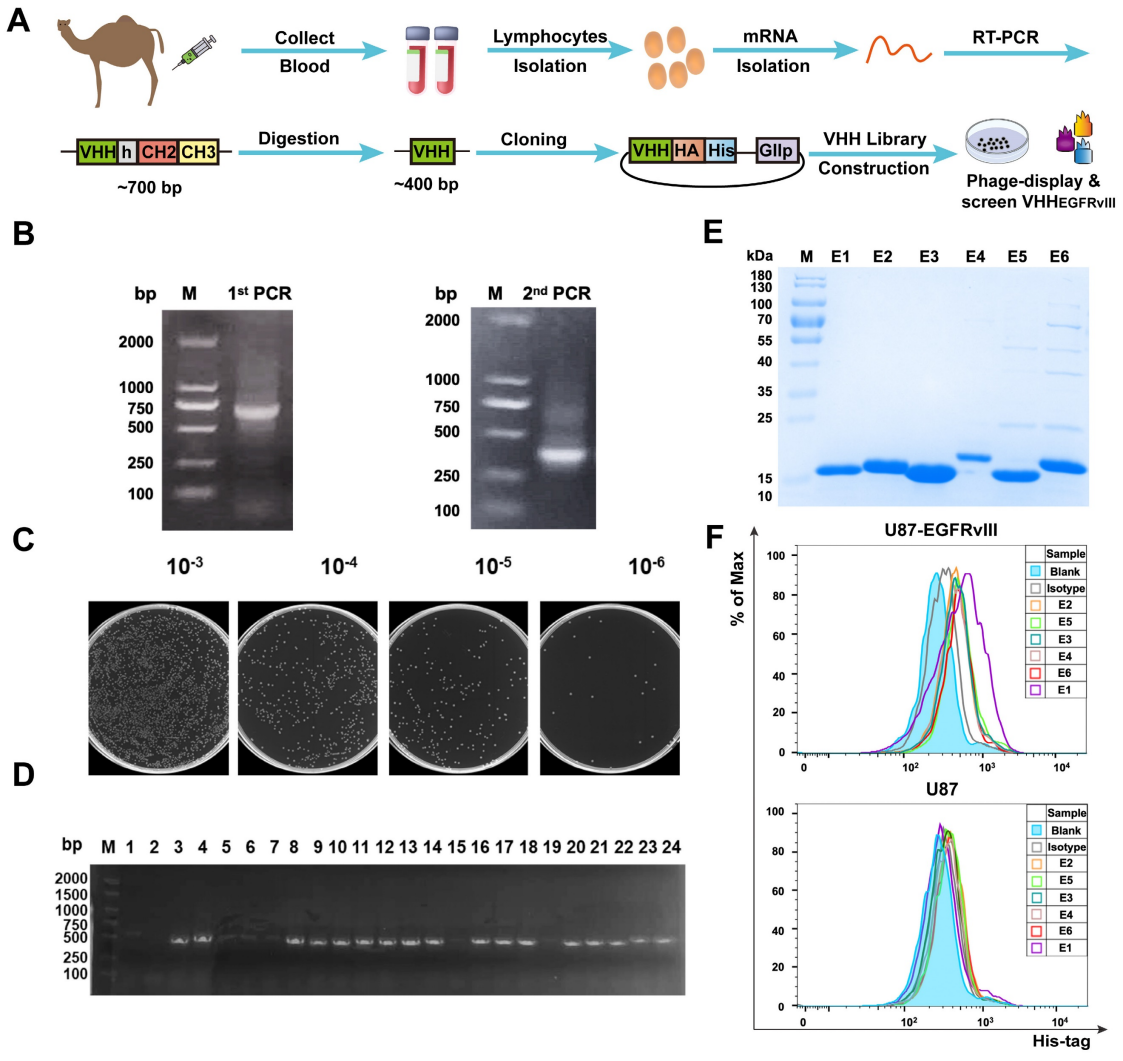
** Library construction and screening of human EGFRvIII nanobody.** (A) Schematic procedure for the acquisition of specific nanobodies from a phage display library. (B) Identification of VHH genes amplification by two steps PCR. (C) The library size was measured by counting the colonies number after serial dilution. (D) Twenty-four colonies were randomly picked to confirm proper insertion rate of VHH genes by PCR amplification. (E) SDS-PAGE analysis of purified EGFRvIII-specific nanobodies (Lane E1-E6), marker (Lane M). (F) Binding ability of anti-EGFRvIII nanobodies to human EGFRvIII^+^ GBM cells (U87-EGFRvIII) and human EGFRvIII^-^ GBM cells (U87) by flow cytometry analysis.

To generate EGFRvIII Nbs with properties of good affinity and specificity. As shown in **Figure 1A**, PBMCs derived from immunized camels were isolated in order to construct the Nb library. **Figures 1B** showed that a ~700 bp band was amplified by the 1^st^ PCR and a ~400 bp band amplified by the 2^nd^ PCR. Then, positive clones were randomly selected and sequenced to assess the quality of the library of 1.3 × 10^8^ clonal forming units (CFU) (**Figure 1C**) and PCR analysis revealed that the library had an insertion rate of 87.5% through randomly selected 24 colonies (**Figure 1D**). Five rounds of bio‑panning were performed for enriching the phages expressing EGFRvIII‑specific Nbs, which were 256‑fold higher than the negative control (**Figure S1A**). These results suggested that this Nb library with good quality and high diversity. Subsequently, 96 colonies were randomly selected for PE‑ELISA. 24 colonies were selected as positive colonies whose binding ratios were >3 (**Figure S1B**), and the sequences of positive colonies were analyzed. Next, His_6_-tagged EGFRvIII Nbs were expressed in prokaryotic system and purified by Ni-NTA affinity column, protein bands of ~15 kDa were detected by sodium dodecyl sulfate polyacrylamide gel electrophoresis (SDS-PAGE) analysis (**Figure 1E**). The flow cytometry analysis of the binding ability of EGFRvIII Nbs to GBM cells demonstrated that 6 selected EGFRvIII Nbs were able to recognize EGFRvIII-expressing GBM cells, but not EGFRvIII-negative U87 cells (**Figure 1F**). These data suggested that selected EGFRvIII Nbs exhibited specific and strong binding to human EGFRvIII expressed on target cancer cells, and SPR analysis showed that EGFRvIII Nb had dissociation rate constant (K_D_) of 2.62 × 10^-7^M to hEGFRvIII, so we choosed EGFRvIII Nb (E1) because it had shown the best performance in binding assay.

### Construction and characteristics of EGFRvIII Nb-CAR‑T cells

**Figure 2:**
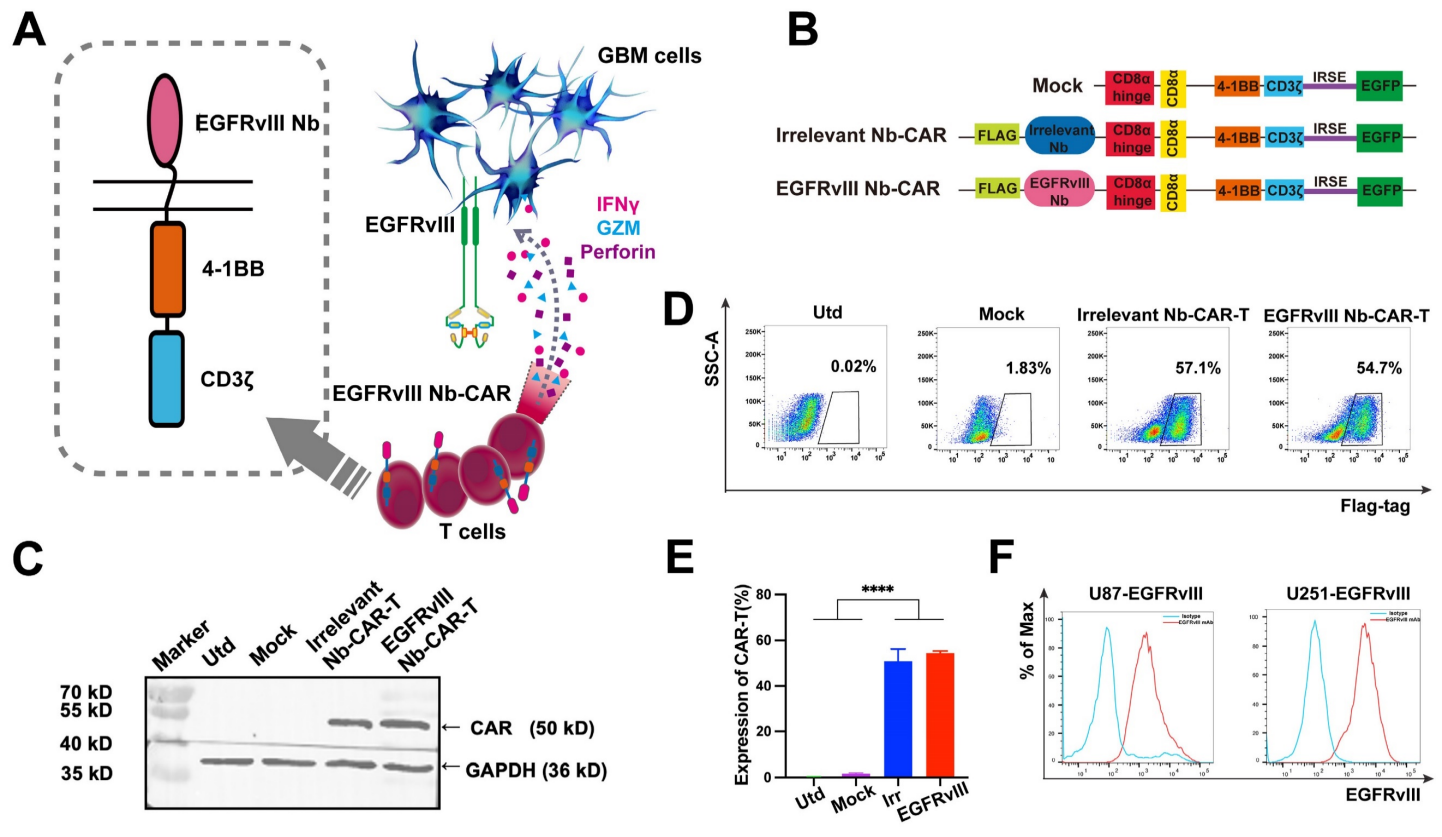
** Generation of EGFRvIII Nanobody-based CAR-T cells.** (A) A working model for therapy with EGFRvIII Nb-CAR-T cells against GBM tumor cells. (B) Schematic diagram of Nb-CAR-T cells. Lentiviral transfer plasmids encoding CARs gene consisting of signal peptide, flag tag, VHH fragments derived from EGFRvIII-specific nanobody, followed by CD8α hinge, transmembrane domain, 4-1BB costimulatory endodomain, and CD3ζ signaling domain along with enhanced green fluorescent protein (EGFP), cDNA separated from the CAR sequence by an internal ribosome entry site (IRES) served as a marker. Utd, Mock, Irrelevant Nb-CAR as control groups. (C) Nb-CARs expression on T cells. Cell lysates were collected and blotted with anti-flag tag antibody and HRP-conjugated IgG antibody as the primary and secondary antibody, then specific bands were observed. (D) Representative pseudocolors showing the transduction rate of PBMC with CARs-encoded lentivirus, detected with APC labeled anti-flag tag antibody by flow cytometry. (E) Graph summarize data from 3 independent measurements, bars represent mean ± SD. (F) EGFRvIII-antigen expressing U87 and U251 cell lines were generated by transduction of EGFRvIII encoding lentivirus. Histogram showed that EGFRvIII overexpression in U251-EGFRvIII and U87-EGFRvIII cells by flow cytometry.

Schematic representation and mechanism of action of EGFRvIII Nb-CAR-T cells recognizing EGFRvIII^+^ target cells and effectively mediating target cell killing effect (**Figure 2A**). A second generation Nb-based CAR specifically targeting EGFRvIII (EGFRvIII Nb-CAR) was constructed, with untransfected (Utd) T, Mock T, Irrelevant Nb-CAR as control groups (**Figure 2B**). We used CAR lentivirus to transduce activated T cells, then western blot analysis showed the full-length CAR with a molecular weight of ~50 kDa was detected in cell lysates using anti-flag tag antibody, and the expected target band sizes in EGFRvIII Nb-CAR‑T cells (**Figure 2C**). The surface expression of EGFRvIII Nb-CAR‑T cells was detected at 3 days post-transduction by flow cytometry. The positive rate of Nb-CAR-T cells was >50% (**Figure 2D**), and histograms showed their quantification (**Figure 2E**). These results indicated that we successfully generated Nb-CAR-T cells for further studies.

### EGFRvIII Nb-CAR‑T cells exert an effector function and specific cytotoxicity* in vitro*

**Figure 3:**
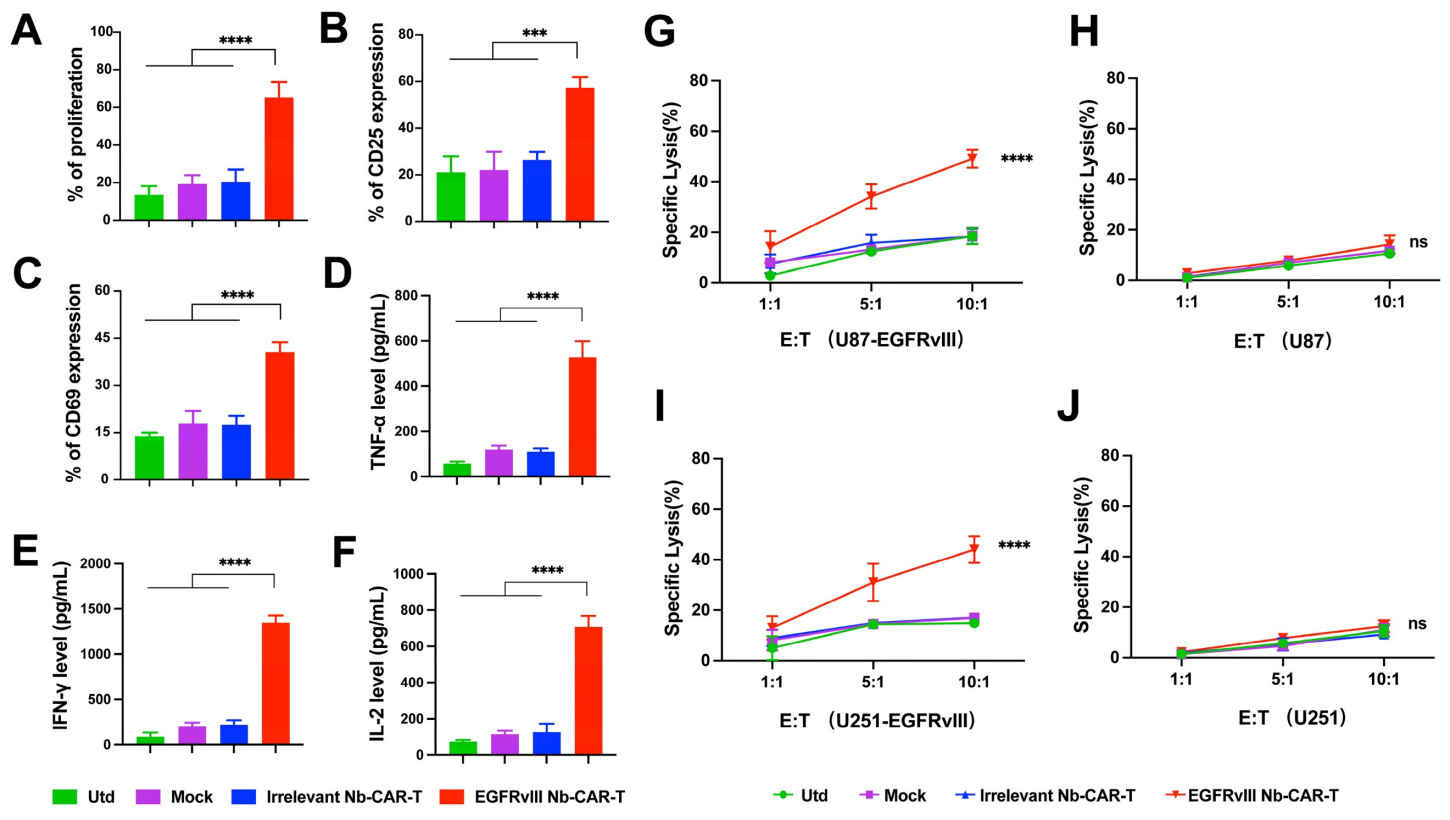
**EGFRvIII Nb-CAR-T cells are capable of proliferation, activation, cytokine production and cytotoxicity *in vitro*.** (A) EGFRvIII Nb-CAR-T cells performed obvious proliferation in response to U251-EGFRvIII. PKH26-labeled EGFRvIII Nb-CAR-T cells were co-incubated with mitomycin C treated-U251-EGFRvIII target cells at an E/T of 1:1 for 4 days and then measured by flow cytometry. (B - C) CD25 and CD69 surface activation marker upregulation was detected 24 h after cocultured with U251-EGFRvIII at an E/T of 1:1 by flow cytometry. (D - F) Cytokine production measured by ELISA in collected cell culture supernatants by Utd, Mock, Irrelevant Nb-CAR-T and EGFRvIII Nb-CAR-T cells when cocultured overnight for 24 h with U251-EGFRvIII at an E/T of 1:1. (G - J) Cytotoxicity of EGFRvIII Nb-CAR-T cells after 16 h co-culture with mitomycin C treated-EGFRvIII^+^ cells and EGFRvIII^-^ cells at different E/T ratios by PKH26^+^7AAD^+^ by flow cytometry. Data are presented as mean values ± SD (n=3).

To determine whether EGFRvIII Nb-CAR‑T cells exert an effector function stimulated by EGFRvIII^+^ target cells *in vitro*. First, we used lentiviral vectors to construct cell lines stably overexpressing EGFRvIII (U87-EGFRvIII and U251-EGFRvIII). Flow cytometry results confirmed EGFRvIII high expression on both U87-EGFRvIII and U251-EGFRvIII cell lines, but not on U87 and U251 (**Figure 2F, Figure S2**). We evaluated proliferation of CAR-T cells with EGFRvIII^+^ target cells U251-EGFRvIII by flow cytometry, results suggested that EGFRvIII Nb-CAR‑T cells showed a very prominent T-cell proliferation response compared to other groups (**Figure 3A**), and a higher CD25 and CD69 T-cell activation expression in EGFRvIII Nb-CAR-T cells than other groups (**Figure 3B and C**), representative histogram was shown in **Figure S3**. These data further indicated that EGFRvIII Nb-CAR‑T cells can exhibit strong T cell function capacities upon stimulation of EGFRvIII^+^ target cells. We found that EGFRvIII Nb-CAR-T cells can respond to the U251-EGFRvIII stimulation with the highest production of pro-inflammatory cytokines such as TNF-α (**Figure 3D**), IFN-γ (**Figure 3E**), and IL-2 (**Figure 3F**), which confirmed that EGFRvIII Nb CAR-T cells were highly activated and proliferated to release cytokines during T-cell mediated immune effectors.

To evaluate the specific cytotoxicity of EGFRvIII Nb-CAR‑T cells against EGFRvIII^+^ target cells *in vitro*, we then incubated EGFRvIII Nb-CAR‑T cells with U87-EGFRvIII, U251-EGFRvIII, U87 and U251 respectively at different effector to target (E/T) ratios. The cytotoxicity of EGFRvIII Nb-CAR‑T cells against EGFRvIII^+^ cells such as U87-EGFRvIII (**Figure 3G**) and U251-EGFRvIII (**Figure 3I**) was significantly higher than other groups by flow cytometry, but no obvious cytotoxic effect on U87 cells (**Figure 3H**) or U251 cells (**Figure 3J**). Thus, these results showed that EGFRvIII Nb-CAR‑T cells possessed specific cytotoxicity against EGFRvIII^+^ cells.

Taken together, these data clearly indicated that EGFRvIII Nb-CAR-T cells strongly upregulate T cell functional markers, significantly enhance T cell proliferation and activation, and profoundly affect proinflammatory cytokine expression* in vitro*, suggesting that antigen specificity of EGFRvIII Nb-CAR‑T cells exert potent target killing efficiency upon stimulation with EGFRvIII^+^ target cells.

### EGFRvIII Nb-CAR‑T cells exhibit efficacious antitumor activity against EGFRvIII^+^ tumors *in vivo*

**Figure 4:**
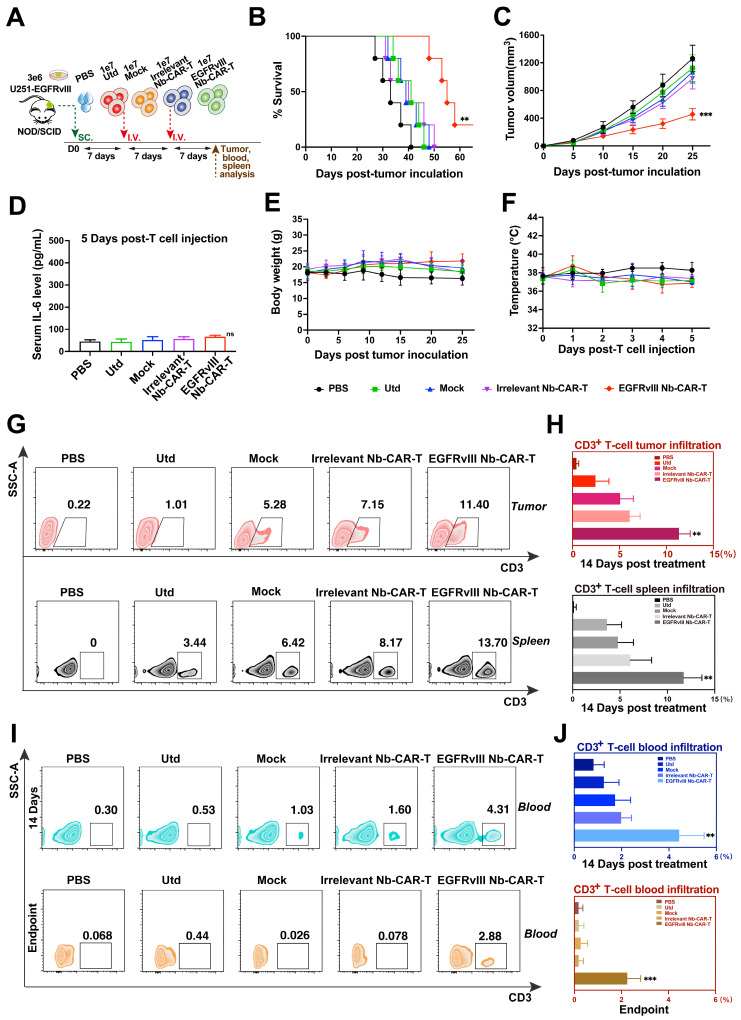
** Antitumor activity, safety and persistence assessment of EGFRvIII Nb-CAR-T cells *in vivo*.** (A) Schematic representation of the *in vivo* mice model and treatment scheme. NOD/SCID mice were inoculated subcutaneously with 3 × 10^6^ U251-EGFRvIII tumor cells and treated with 1 × 10^7^ EGFRvIII Nb-CAR-T, Irrelevant Nb-CAR-T, Mock, Utd cells and PBS as control. Survival ratio curve (B) and tumor volume (C) were monitored of each treatment cohort to access efficacy (n=5 per group). (D) Serum cytokine level of IL-6 were measured using ELISA on day 5 after EGFRvIII Nb-CAR T-cells transfer. Body weight (E) and fever measurements (F) demonstrate no gross toxicity. (G) Representative flow plots showing the frequency of EGFRvIII Nb-CAR-T cells in tumor issue and spleen of treated mice at day 14 after EGFRvIII Nb-CAR-T cells infusion, summarized results are shown in (H). (I) Representative flow plots showing the frequency of EGFRvIII Nb-CAR-T cells in blood of treated mice at day 14 and endpoint after CAR-T cells infusion, summarized results are shown in (J). Data are presented as mean values ± SD.

We then assessed their antitumor activity in mouse GBM models. As shown in **Figure 4A**, NOD/SCID mice were injected subcutaneously with 3 × 10^6^ U251-EGFRvIII tumor cells, after 7 days, mice were treated with 1 × 10^7^ EGFRvIII Nb-CAR‑T cells by tail vein injection. The results showed that EGFRvIII Nb-CAR‑T cells were more effective in prolonging the survival of tumor-bearing mice compared to other groups (**Figure 4B**). We also observed that tumor growth in mice was significantly inhibited after injection with EGFRvIII Nb-CAR‑T cells, while tumor size in other groups continued to grow rapidly without obvious antitumor effects (**Figure 4C**). Moreover, EGFRvIII Nb-CAR‑T cells group was well controlled for tumor progression and prolonged survival in U87-EGFRvIII subcutaneous tumor model of NOD/SCID mice (**Figure S4A-C**). Particularly, there was no significant toxic effect throughout the whole* in vivo* experiments, the IL-6 level (**Figure 4D**), body weight (**Figure 4E**) and temperature (**Figure 4F**) also had no obvious changes after treatment. Together, these results showed that the therapeutic potential and safety of EGFRvIII Nb-CAR‑T cells.

We further investigated the antitumor mechanism of EGFRvIII Nb-CAR-T cells *in vivo* by measuring the presence of EGFRvIII Nb-CAR-T cell infiltration in tumor tissues, blood and spleen of NOD/SCID mice. As shown in **Figure 4G**, flow cytometry analysis revealed that at day 14 after administration, the percentage of CAR-T cells in both tumor tissue and spleen was significantly higher in EGFRvIII Nb-CAR‑T cells treated mice than that in the other groups. Moreover, the presence of CAR-T cells was still detectable in peripheral blood in treatment groups until endpoint after EGFRvIII Nb-CAR-T cells injection, slightly reduced when compared to 14 days, but Nb-CAR-T cells in blood were significantly higher than in other groups (**Figure 4I-J**). Therefore, these data demonstrated that preferable antitumor efficacy may owing to the increased infiltration and persistence of EGFRvIII Nb-CAR‑T cells *in vivo*.

### Characterization of the DC/tumor fusion vaccines

**Figure 5:**
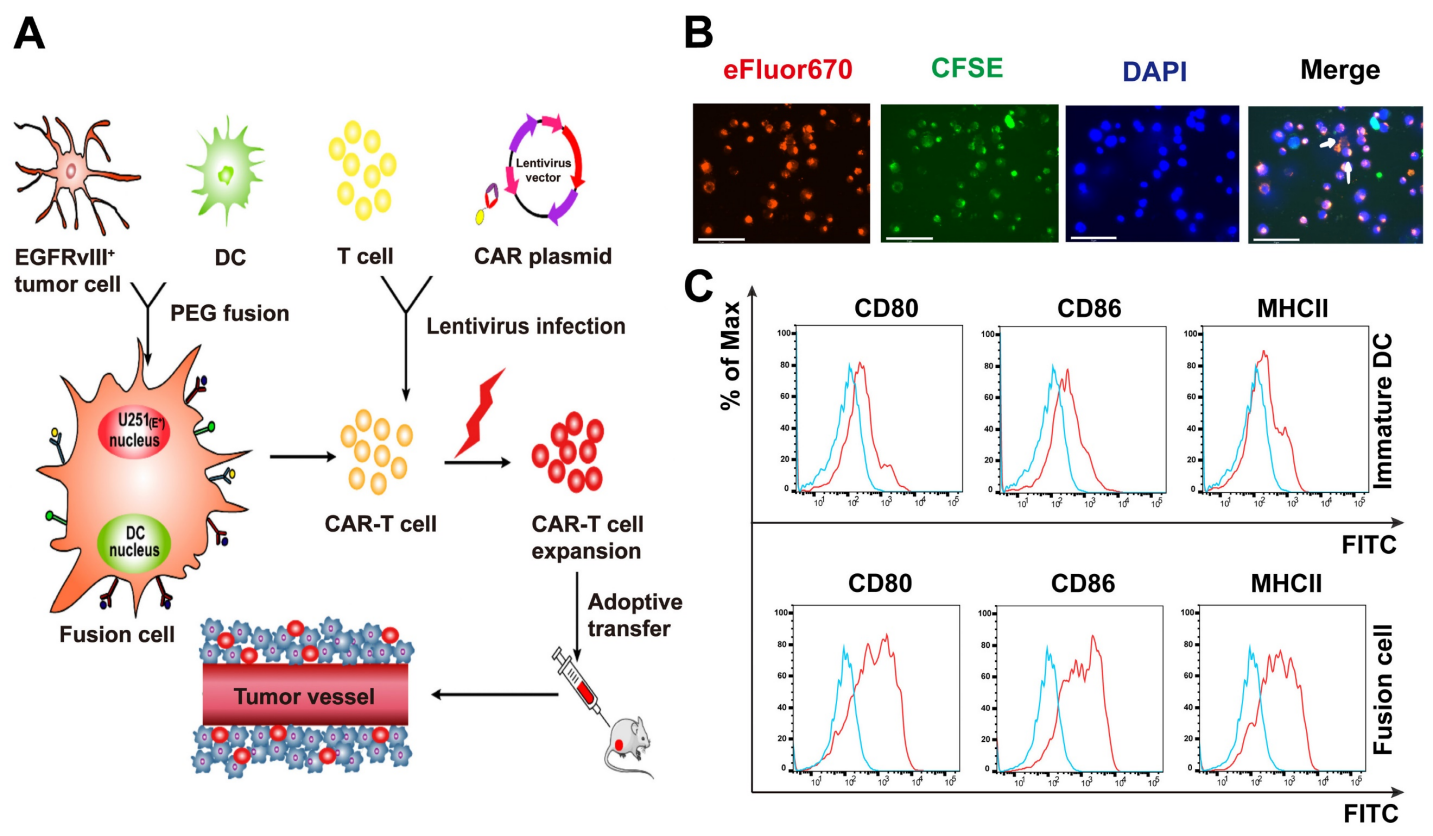
**Characterization and identification of DC/GBM fusion cells**. (A) Schematic diagram of antitumor mechanism of Nb-CAR-T cells promoted by DC/tumor fusion cell vaccines. (B) Autologous DCs were generated from adherent mononuclear cells isolated from a leukapheresis collection, DCs and tumor cells were labeled with CFSE (green) and eFluor670 (red) respectively, followed by fusion with PEG. Subsequently, the cells were stained with DAPI (blue) and observed under a fluorescent microscope (scale bar, 75 μm). The white arrow indicates fusion cells. (C) Fusion cells were analyzed for expression of costimulatory molecules CD80, CD86, MHCII compared with immature DCs by flow cytometry.

Schematic diagram of the antitumor mechanism of Nb-CAR-T cells promoted by tumor/DC fusion cell vaccines was shown in **Figure 5A**. CFSE-labeled DCs were fused with eFlour670-labeled EGFRvIII^+^ GBM cells using polyethylene glycol (PEG). We observed that CFSE-labeled tumor cells (green) were fused with eFluor670-labeled DCs (red), the cells were stained with DAPI (blue) under a fluorescent microscope (**Figure 5B**), white arrows represented FC cells that overlapped the two colors of fluorescent markers, which showed the FC cells were successfully fused, it was revealed that FCs conserved the ability of DCs to respond to triggering stimulatory factors and may have a strong antigen presentation capacity that allows Nb-CAR-T cells to be activated in response to antigens. A significant upregulation of CD80, CD86 and MHCII molecules was observed on FC in comparison with the immature DCs (**Figure 5C**). Therefore, FCs could serve as antigen-presenting cells to further stimulate Nb-CAR-T cells proliferation and function.

### DC/tumor fusion vaccines enhance the anti-tumor function of EGFRvIII Nb-CAR-T cells *in vitro.*

**Figure 6:**
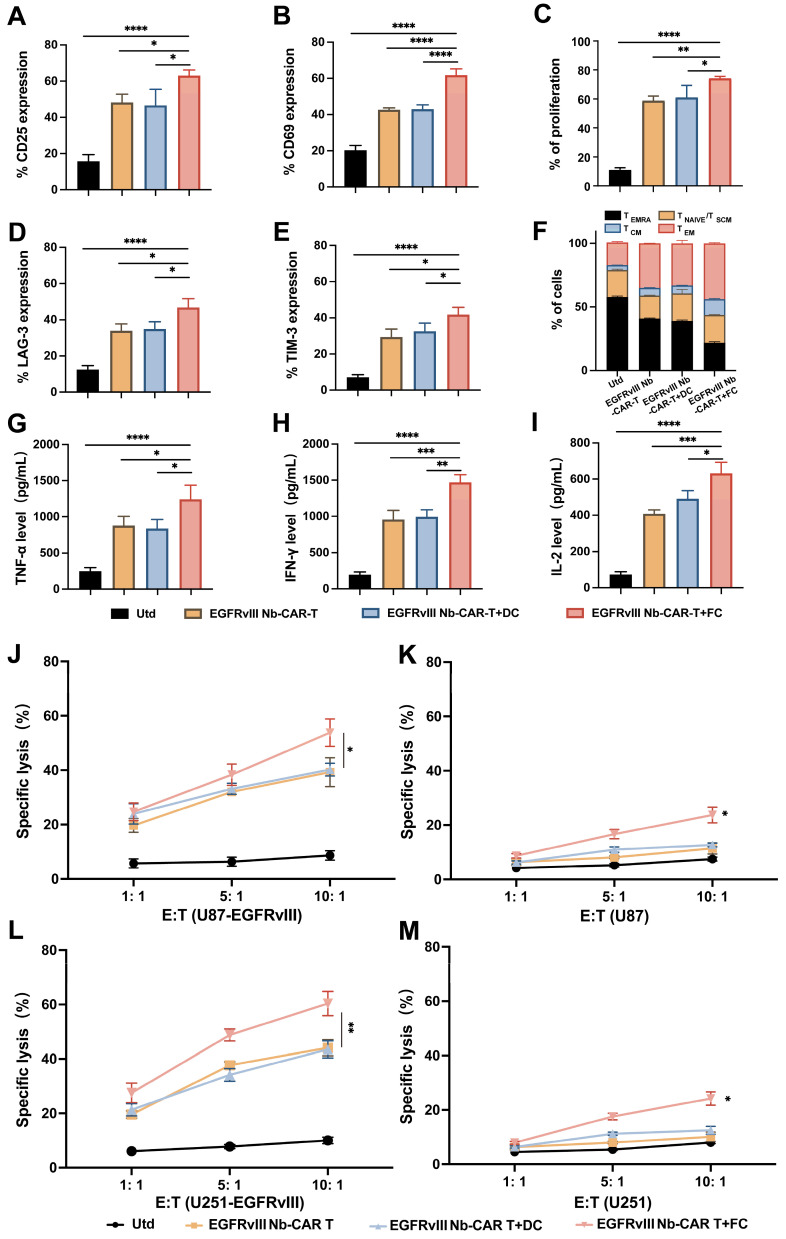
** DC/tumor fusion vaccines enhance EGFRvIII Nb-CAR-T effector function and cytotoxicity *in vitro*.** (A - B) After coculture with U87-EGFRvIII cells at an E/T of 1:1, EGFRvIII Nb-CAR-T cells stimulated with FCs showed more increased expression of CD25, CD69 compared to other groups. (C) eFluor670 dilution was measured by flow cytometry 4 days later and the percentages of divided effector cells was shown. (D - E) T cell exhaustion of LAG-3 and TIM-3 expression increased in EGFRvIII Nb-CAR-T+FC compared to other groups after cocultured with U87-EGFRvIII for 3 days at an E/T of 1:1. (F) After 14 days, CD45RA^-^ / CD62L^+^ as TCM cells was higher in EGFRvIII Nb-CAR-T+FC group than other groups. (G - I) Cytokine levels by ELISA in collected cell culture supernatants when cocultured with U87-EGFRvIII for 24 h at an E/T of 1:1. (J - M) Cytotoxicity of EGFRvIII Nb-CAR-T+FC cells after 16 h coculture with target cells at different E/T ratios by eFluor670^+^7AAD^+^ double staining and flow cytometry. All representative of 3 independent experiments, Data are presented as mean values ± SD.

To demonstrate whether DC/tumor fusion vaccines enhance the antitumor efficacy of EGFRvIII Nb-CAR-T cells and in association with the activation of CAR-T cells targeting specific antigens, we also investigated the memory and exhaustion phenotype of CAR-T cells after EGFRvIII Nb-CAR-T cells and FCs were cocultured with U87-EGFRvIII cells. These data showed that the expression of CD25 (**Figure 6A**), CD69 (**Figure 6B**) and T cell proliferation (**Figure 6C**) were significantly increased compared with Utd, EGFRvIII Nb-CAR-T, EGFRvIII Nb-CAR-T+DC groups. We also observed that with DC/tumor fusion vaccines stimulation, EGFRvIII Nb-CAR-T cells had more acquired expression of LAG-3 (**Figure 6D**), TIM-3 (**Figure 6E**) compared to other groups. We further used expression of CD62L and CD45RA to define T cell memory subsets, and percentage of central memory T cells (TCM, CD45RA^-^/CD62L^+^) in EGFRvIII Nb-CAR-T+FC group was higher than other groups. Representative histograms of flow cytometry are found in **Figure S5**. Overall, our results revealed that DC/tumor fusion vaccines stimulated to enhance EGFRvIII Nb-CAR-T cells function, proliferation, central-memory-like differentiation and ultimately exhaustion, probably due to FCs could possess mighty capacity of target antigen presentation.

It is known that T cells secrete a number of cytokines to support a cellular immune response and kill tumor cells. We also observed that the highest levels of TNF-α (**Figure 6G**), IFN-γ (**Figure 6H**), and IL-2 (**Figure 6I**) in EGFRvIII Nb-CAR-T+FC group, while there was not much difference between EGFRvIII Nb-CAR-T and EGFRvIII Nb-CAR-T+DC groups. These results may explain that FCs enhance the antigen presentation of DCs and activate CAR-T cells more effectively.

Next, we found that DC/tumor fusion vaccines increased the cytotoxicity of EGFRvIII Nb-CAR-T cells *in vitro*. The results suggested that EGFRvIII Nb-CAR-T cells induced by DC/tumor fusion vaccines possessed significantly cytotoxicity against EGFRvIII^+^ cells U87-EGFRvIII (**Figure 6J**) and U251-EGFRvIII (**Figure 6L**) than other groups. Intriguingly, the cytotoxicity against was not only EGFRvIII^+^ cells but also EGFRvIII^-^ cells U87 (**Figure 6K**) and U251 (**Figure 6M**), when at the E:T ratio of 10:1, EGFRvIII Nb-CAR-T+FCs had a certain effect on tumor cell killing against EGFRvIII^-^ cells. Therefore, these data revealed that EGFRvIII Nb-CAR-T cells stimulated by DC/tumor fusion vaccines resulted in increased cytotoxicity and efficiency *in vitro*. Furthermore, FCs induced antigen-specific killing of Nb-CAR-T cells was not restricted to the target‐positive cells also able to other potential antigen-expressing target cells.

### DC/tumor fusion vaccines enhance EGFRvIII Nb-CAR-T antitumor efficiency *in vivo*

**Figure 7:**
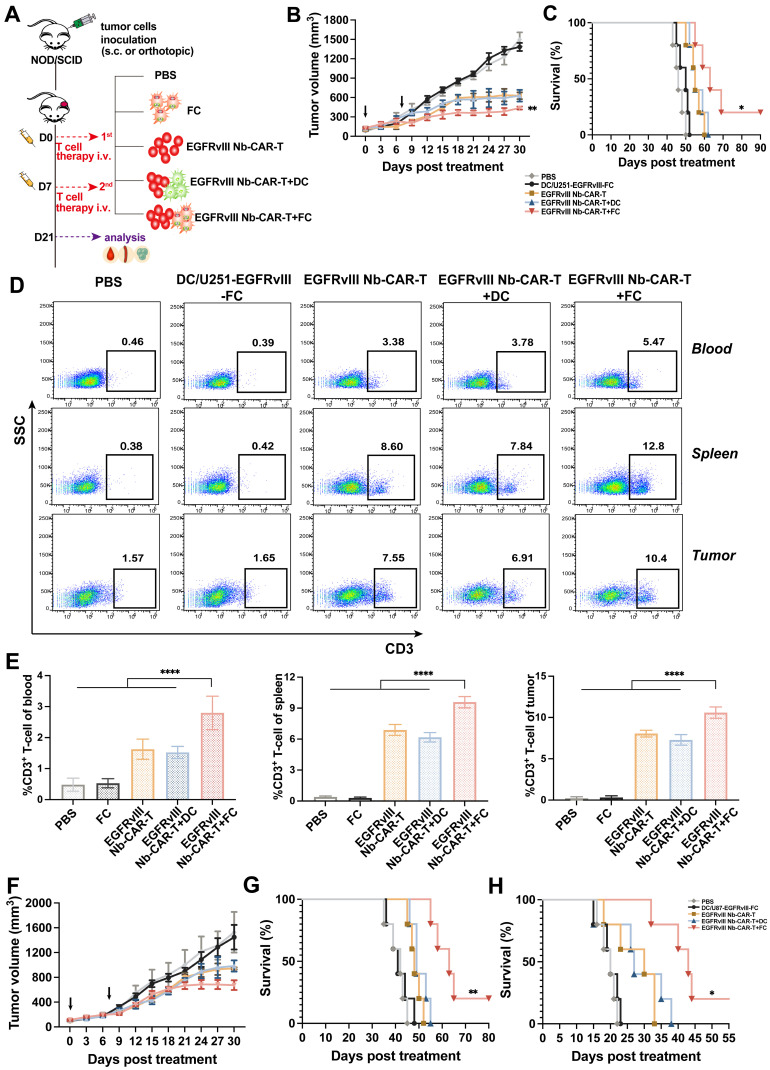
** DC/tumor fusion vaccines enhance antitumor effects of EGFRvIII Nb-CAR-T cells in xenograft mice models.** (A) Schematic representation of *in vivo* mice model and treatment scheme. (B - C) NOD/SCID mice were subcutaneously inoculated with 3 × 10^6^ U251-EGFRvIII tumor cells and treated with 1 × 10^7^ DC/U251-EGFRvIII-FC, EGFRvIII Nb-CAR-T, EGFRvIII Nb-CAR-T+DC, EGFRvIII Nb-CAR-T+FC and PBS as control groups. Tumor volume and survival ratio curve were recorded (n=5). (D - E) Representative flow plots showing the more persistence of CAR-T cells in tumor issue, spleen and blood of treated mice at 14 d after injection of EGFRvIII Nb-CAR-T+FCs. (F - G) Similarly, NOD/SCID mice were inoculated subcutaneously with 3 × 10^6^ U87-EGFRvIII tumor cells and treated as above. Tumor volume and survival ratio curve were recorded (n=5). (H) Survival curves for *in vivo* mouse orthotopic U87-EGFRvIII GBM model. Data are presented as mean values ± SD.

We proceeded to evaluate the therapeutic potential of EGFRvIII Nb-CAR-T cells induced by DC/tumor fusion vaccines by subcutaneously inoculating NOD/SCID mice with U251-EGFRvIII or U87-EGFRvIII tumor cells as solid tumor models. *In vivo* results showed that EGFRvIII Nb-CAR-T+FCs therapy group significantly prolonged recipient survival and delayed tumor growth both in U251-EGFRvIII (**Figure 7B-C**) and U87-EGFRvIII (**Figure 7F-G**) tumor-bearing mice models compared with PBS, FCs, EGFRvIII Nb-CAR-T cells and EGFRvIII Nb-CAR-T+DCs groups. Moreover, Nb-CAR-T cells infiltration in tumor tissue, spleen and blood was analyzed at 14 days after treatment, we observed that EGFRvIII Nb-CAR-T+FCs showed improved persistence *in vivo*, results revealed that there was a significant increase in the percentage of CAR-T cells upon EGFRvIII Nb-CAR-T+FC treatment in comparison to other groups in U251-EGFRvIII tumor bearing mice (**Figure 7D-E**). Similarly, we further confirmed these findings in another U87-EGFRvIII subcutaneous tumor model in NOD/SCID mice (**Figure S6A-B**). In order to better simulate the tumor environment, we performed stereotactic orthotopic injection of U87-EGFRvIII cells, the result demonstrated that the survival time was significantly prolonged in the EGFRvIII Nb-CAR-T+FCs treatment group (**Figure 7H**). Taken together, these data indicated that FCs with EGFRvIII Nb-CAR-T cells allowed for higher responses *in vivo* by inducing substantially stronger effects of Nb-CAR-T cells targeting specific tumor antigens.

## Discussion

CAR-T cell-based adoptive cellular immunotherapy has made great progress on hematological malignancy but establishing these treatments for solid tumors has been difficult. This is primarily owing to lack of homogenously expressed or specific candidate targets to direct effector T cells against the tumor cells and an antagonistic immunosuppressive solid tumor microenvironment 37. It is urgently needed for discovering specific markers and establishing reliable and effective CAR-T cells available to accumulate and penetrate into the tumor tissues for exploiting CAR-T cells to solid tumor treatments. Our study has innovatively excogitated a high-specific Nb against the EGFRvIII, then sharing with the promising data firstly on this EGFRvIII Nb-CAR-T cells improve antitumor activity primed by the DC/tumor fusion vaccines in a preclinical mouse model. In this study, we hypothesized that the new Nb-CAR-T cells stimulated with DC/tumor fusion vaccines could enhance their antitumor efficacy for solid tumor treatment. Our results demonstrated that nanobody-based CAR-T cells were readily expanded with stimulation of DC/tumor fusion vaccines as a more tumor-reactive CAR-T phenotype in the mice models. To our knowledge, the present report is the first one to conceptually present the elements on that an antitumor efficacy of nanobody-based CAR-T cells targeting tumor-specific antigens is significantly boosted by DC/tumor fusion vaccines *in vivo* and* in vitro* study.

Firstly, nanobodies were generated by immunizing a camel with human EGFRvIII recombinant proteins and using routine phage display technology, which enriched and selected hits from our successful construction of immune nanobody phage display libraries by five rounds of panning against the target antigen. In this study, we developed EGFRvIII Nbs specifically to bind epitopes, which may be different from traditional EGFRvIII antigen epitopes targeted by scFvs. Furthermore, we verified the high binding affinity of 6 Nbs to EGFRvIII-expressing cells by flow cytometry and selected the good binding affinity EGFRvIII Nb (E1) for our further investigation. Moreover, SPR revealed that the dissociation rate constant (K_D_) of EGFRvIII Nb was 2.62 × 10^-7^M, which was consistent with previous literature 38. Therefore, Nbs, grant the exceptional edge in low-cost industrialization production and simplified prokaryotic expression compared to scFvs, which not only has the potential for fast clinical translation but also provides an invaluable tool in tumor immunotherapy as well as many other areas of research 39,40.

Based on immune phage library screening and identification of good binding affinity EGFRvIII Nb, which was applied to another new design idea to make a kind of EGFRvIII Nb-based CAR-T cells via lentiviral infection technology. The infection efficiency of EGFRvIII Nb-CAR-T cells demonstrated successful Nb-CAR-T cells preparation. Furthermore, cytokine release and cytotoxic killing assay confirmed that Nb-CAR-T cells had the potent antitumor effect *in vitro* and persistent CAR-T cells infiltration in xenograft GBM mouse models, without obvious side effects. Consequently, we screened specific EGFRvIII Nb and validated the antitumor efficiency of EGFRvIII Nb-CAR-T cells for the first time. We noted that the rapid antigen recognition of CAR-T cell-compatible Nb may allow an adequate response and quick expansion of CAR-T cells by overcoming the limited efficacy of conventional scFv-derived CAR for its misfolded heavy-light chains 41. This report suggests that arming with nanobody enables CAR-T cell therapy to target universal antigens on lung cancer, liver cancer, ovarian cancer and another solid diseases, providing a promising platform for ACT strategies and delivering a long-lasting CAR-T cells product to patients.

The most important idea presented here is to apply the new strategy of DC/tumor fusion vaccines stimulating Nb-CAR-T cells efficacy. DC/tumor fusion vaccines are intended to recruit tumor-specific T cells for motivating immunological memory and hindering existing tumor recidivation 42-45. From a phase I trial of DC/myeloma fusions, vaccination was proved well tolerated, inducing anti-myeloma immunity in late-stage patients and resulting in prolonged disease stability 46. The DC/tumor fusion cell vaccine previously constructed by our group has shown certain advantages in antitumor immunotherapy. For example, CD8^+^ T cells pretreated with PD-1 Nb20 and DC/tumor cell fusion cell vaccines significantly inhibited the growth of NSCLC, HCC, and TSCC xenografts, enhanced T cell infiltration into the tumor tissues and inhibited tumor angiogenesis, which provided an alternative immunotherapy strategy for tumor patients with or without T cell dysfunction 35. Herein, the fusion cell vaccine has inherited the antigen presentation ability of DCs, loaded the known versus the unknown tumor antigens, and presented multiple epitopes to Nb-CAR-T cells recognition, resulting in more effective activation and proliferation of Nb-CAR-T cells. Interestingly, CD45RA^-^ / CD62L^+^ expression demonstrated that EGFRvIII Nb-CAR-T+FCs improved the antitumor efficacy more significantly, as a result of differentiation into central memory T cells 47. TIM-3 and LAG-3 expression increased significantly with EGFRvIII Nb-CAR-T+FCs, which is consistent with up-regulated preferentially in stimulating T cell activation to limit exaggerated responses and potential tissue damage 48. We found that FCs significantly enhanced EGFRvIII Nb-CAR-T cells immunotherapy in several xenograft GBM mouse models, provided from extended survival and tumor regression, incremental CAR-T cells infiltration and persistence in the solid tumor tissues as well. One possible mechanism is that FCs cross-presentation captures antigen and migrates to regional tumor lymph nodes, where T cells are activated and accumulated, may speed up further extension of infiltrated Nb-CAR-T cells and blasting of multiple stromata in solid tumors. On the other hand, FCs may induce immune responses and prime durable antigen stimulation upon CAR-redirected T lymphocytes antitumor activity 49,50 and replenish tumor-recognizing non-CAR-T cells for more available antitumor responses. However, one limitation is that the intermediate mechanism has not been fully investigated, and further work will require humanized PDX models and validate the role of underlying pathways as a general mechanism as well.

Altogether, this study has confirmed our hypothesis that EGFRvIII Nb-based CAR-T cells are stimulated by DC/GBM fusion vaccines for solid tumor treatments. This appears a new kind of pattern and strategy for rescuing Nb-CAR-T cells activity upon stimulation with DC-derived tumor fusion vaccines. Our current findings may also constitute an important step for Nb-CAR-T cells penetrating into deep solid tumors. Furthermore, this idea may be similarly applied to other T cells that are activated by any agents, such as combination with bispecific T cell engagers (BiTEs) and immune checkpoint blockade (ICB) to further enhance T cell antitumor responses in future studies.

In summary, this first preclinical study has proved an improved therapeutic effect of DC/tumor fusion vaccines enhancing Nb-CAR-T cells antitumor effect in solid tumor treatments. We believe that our design concept can be applied to many other cellular and intracellular antigens, and other nascent joint strategies for TAA-targeted T cell engager and FC vaccines. Therefore, the Nb-based CAR design with DC/tumor fusion cell vaccination offers a simple and universal solution to enhancing therapeutic efficacy of any antigen specificity Nb-CAR-T cells for solid tumors. This new strategy provides guidance on direct implications for the combination design of future adoptive cellular therapy for solid tumor treatments.

## Materials and Methods

### Cell lines and culture

Human GBM cells U87 and U251 were acquired from Meisen CTCC. They were cultured according to manufacturer's recommendations. The EGFRvIII overexpressing U87 and U251 cell lines were generated by selecting the cells that were stably transfected with hEGFRvIII lentivirus. U87, U251, U87-EGFRvIII, U251-EGFRvIII cells were maintained in 10% FBS (Gibco, USA) complete DMEM with 100 U/mL penicillin - streptomycin (Solarbio, China) at 37 °C in 5% CO_2_.

Peripheral blood mononuclear cells (PBMCs) were collected from healthy human donors, then were separated through Ficoll density gradient centrifugation. Nonadherent cells were resuspended in 10% FBS complete RPMI 1640 media with 100 U/mL rhIL-2 added. Experiments including human samples were approved by the Institutional Review Board of Guangxi Medical University (Guangxi, China).

### Generation and characteristic of Nanobody

EGFRvIII Nbs were screened by phage display technology according to our previously described 35,51. EGFRvIII Nbs with His_6_ tagged were purified using Ni-NTA affinity columns. The specific purified EGFRvIII Nbs were analyzed by SDS-PAGE. For EGFRvIII Nbs specific binding assay, EGFRvIII Nbs were incubated with corresponding target cells for 30 min, then cells were stained with APC labeled anti-his tag antibody. The binding was detected by flow cytometry on a FACS Canto analyzer (BD Biosciences, USA).

### Nb-CAR lentiviral design and construction

For EGFRvIII Nb-CAR, the EGFRvIII Nb domain was incorporated into the 2^nd^ basic generation CAR, which consisted of signal peptide, CD8α hinge, CD8 transmembrane domain, and 4-1BB costimulatory signal and CD3ζ activation signal, and enhanced green fluorescent protein (EGFP) tracer for following transduction and flag tag for subsequent analysis. EGFRvIII Nb-CAR constructs were cloned into GV538 lentiviral vector and were validated by sequencing (provided by GeneChem Inc., China), Mock-T and Irrelevant Nb-CAR-T were generated for the control groups. Lentiviral EGFRvIII Nb-CAR was generated by co-transfections of HEK293T cells and then harvested supernatants containing lentiviral particles were assayed for titer determination.

### Generation of DC/tumor fusion vaccines

DC isolation and fusion techniques are following our previous experimental protocols 34,35. Briefly, DCs were isolated from the adherent fraction of PBMC and cultured in above medium for 6 days (1000 U/mL rhGM-CSF and 500 U/mL rhIL-4) and further cultured for 2 days (500 U/mL rhTNF-α), cytokines were replenished every other day. rhGM-CSF, rhIL-4, rhTNF-α were all purchased from Peprotech, USA. For evaluating the fusion efficiency, eFluor670-labeled EGFRvIII^+^ tumor cells were incubated with CFSE-labeled DCs, all cell nuclei were identified with DAPI, FCs were generated using polyethylene glycol (PEG) (Sigma, USA) with heat shock, then slow centrifugation to increase the success of conjugate formation and observed by fluorescence microscopy, FCs were confirmed using FITC-CD80, FITC-CD86, and FITC-MHC-II antibodies (eBioscience, USA).

### Lentiviral transduction of Nb-CAR-expressing human T cells

To prepare Nb-CAR-T cells, primary T cells were stimulated with CD3 mAb and CD28 mAb at a ratio of 2: 1 (eBiosciences, USA) in the media 48 hours prior to transduction. Lentiviral transduction for downstream experiments was accomplished at 10 MOI. After expansion, Nb-CAR-T cells were harvested and transfection efficiency was evaluated by flow cytometry.

### Western blot analysis

Briefly, Nb-CAR-T cells were collected and washed with PBS, then lysed in 1× RIPA lysis buffer (Solarbio, China), samples were then prepared by adding 5× loading buffer to boiling. After SDS-PAGE and wet transfer to polyvinylidene fluoride (PVDF) membrane, and blocked with 5% skim milk, and then incubated with mouse anti-flag tag antibody (1:1000, Invitrogen, USA). After washing, the membrane was incubated with anti-mouse IgG-HRP antibody (1:1000, Abcam, USA) according to the manufacturer's instructions. Protein bands were visualized using BeyoECL Plus chemiluminescence kit (Beyotime, China) and were imaged using the Chemi Doc imaging system (Bio-Rad, USA).

### Nb-CAR-T cell functional phenotype

To assess the influence of EGFRvIII antigen-dependent activation, proliferation, differentiation and exhaustion on T cell phenotypes. Briefly, after coincubation EGFRvIII Nb-CAR-T cells with the target EGFRvIII^+^ cells, T cells surface phenotypes were assessed using the following antibodies: PE or APC anti-human CD25 antibody (BioLegend, USA), PE or APC anti-human CD69 antibody (BioLegend, USA) for T cell activation; PKH26 (Sigma Aldrich, USA) or eFluor670 dye (ThermoFisher, USA) for T cell proliferation; APC anti-human CD62L antibody (BioLegend, USA) and PE-cy7 anti-human CD45RA antibody (BioLegend, USA) for T cell memory function; APC anti-human LAG-3 antibody (BioLegend, USA) and APC anti-human TIM-3 antibody (BioLegend, USA) for T cell exhaustion, all flow cytometry data was analyzed using FlowJo software.

### Enzyme-linked immunosorbent assay (ELISA)

EGFRvIII Nb-CAR-T cells were cocultured with EGFRvIII^+^ cells for 24 h, and then we collected supernatants to measure the levels of inflammatory factors using the Human TNF-α, IFN-γ and IL-2 ELISA Kit (Lianke, China) following the kit instructions. OD_450_ nm were measured in a microplate reader (Tecan, Switzerland). When EGFRvIII Nb-CAR-T cells were cocultured with FCs for 24 h, supernatants we collected and level of cytokines were quantified as described above.

### Cytotoxicity assay

The cytotoxicity of EGFRvIII Nb-CAR-T cells* in vitro* was analyzed with flow cytometry assay. Target cells (U87, U251, U87-EGFRvIII, U251-EGFRvIII) were mitomycin C-treated and labeled with PKH26. They were then added to Nb-CAR-T cells at an E:T ratio of 1:1, 5:1 or 10:1. Then cells were stained with 7-AAD (AAT Bioquest, USA), and cocultured for 16 h at 37°C and 5%CO_2_. The % PKH26^+^PI^+^ dead cells in all groups were calculated by flow cytometry. Additionally, the cytotoxicity induced by FCs was stimulated with Nb-CAR-T cells and cocultured with eFluor670-labeled tumor cells for 16 h, they were then washed and stained with 7-AAD. The % eFluor670^+^7AAD^+^ dead cells were determined by flow cytometry as above. % Specific lysis = (fluorescence (sample lysis) - fluorescence (spontaneous lysis))/(100 - fluorescence (spontaneous lysis)) × 100.

### *In vivo* experiments

All animal experiments were accomplished in accordance with all associated ethical regulations and in conformity to Institutional Animal Care and Use Committee (IACUC) of Guangxi Medical University. For tumor-bearing mouse models, NOD/SCID mice of 4-6 weeks (Beijing Vital River, China) were injected subcutaneously with 3 × 10^6^ U87-EGFRvIII or 3 × 10^6^ U251-EGFRvIII tumor cells respectively. For the orthotopic GBM tumor model, NOD/SCID mice were stereotactically inoculated with U87-EGFRvIII cells, A total of 5 × 10^5^ cells in a volume of 5 μL were injected into the brain region at a rate of 1 μL/min using a mouse brain localizer as previously described 52. After 7 days, the mice were treated with 1 × 10^7^ Nb-CAR-T cells via caudal vein injection once every 7 days for 2 times. For FCs vaccines administration, tumor‐forming mice were treated with 1 × 10^7^ Nb-CAR-T cells with FCs immediately at 7-day intervals for a total of 2 doses. Experimental NOD/SCID mice were closely monitored for physical symptoms throughout their survival. Emaciated animals (i.e., moribund status or agonal breathing pattern) were euthanized. Tumor volumes were calculated as 1/2 × L × W^2^, where W is width and L is length. Similarly, the survival of experimental mice was also be evaluated and the body weight and temperature were monitored at the indicated time. Moreover, after 5 days Nb-CAR-T cells injection, IL-6 levels of serum samples were measured by ELISA. Blood, spleen, and tumor homogenates were prepared for subsequently stained anti-human CD3 antibody (Biolegend, USA) to access the infiltration and persistence of Nb-CAR-T cells by flow cytometry.

### Statistical analysis

GraphPad Prism 9.0 software was applied for statistical analysis. Data represent mean ± SD. We used Kaplan-Meier survival curves and a log rank comparison of the groups to calculate p-values. Student's t-test was conducted for comparison between two groups, one-way analysis of variance (ANOVA) was applied for the comparison of three or more groups, A *p*-value of < 0.05 was regarded as statistically significant. ^*^ *P* < 0.05, ^**^ *P* < 0.01, ^***^ *P* < 0.001 and ^****^ *P* < 0.0001.

## Supplementary Material

Supplementary figures.

## Abbreviations

CAR: chimeric antigen receptor
CAR-T: chimeric antigen receptor-T cell
Nb: nanobody
EGFRvIII: epidermal growth factor receptor variant type Ⅲ
GBM: glioblastoma
VHH: variable domain of heavy chain of heavy-chain antibody
scFv: single-chain variable fragment
FDA: Food and Drug Administration
APC: antigen-presenting cell
CTL: cytotoxic T lymphocytes
7-AAD: 7-amino-actinomycin D
CFU: clonal forming units
FBS: fetal bovine serum
mAb: monclone antibody
DC: Dendritic cell
PKH26: Paul Karl Horan 26
CSFE: carboxyfluorescein succinimidyl amino ester
PBMC: peripheral blood mononucleated cell
PBS: phosphate buffer saline
PCR: polymerase chain reaction
PEG: polyethylene glycol
DAPI: destination access point identifier
FC: Fusion cell
ELISA: enzyme linked immunosorbent assay
GM-CSF: granulocyte macrophage-colony stimulating factor
TNF-α: tumor necrosis factor-α
IL-4: Interleukin-4
IL-6: Interleukin-6
WB: western blotting
NOD/SCID: non-obese diabetic/server combined immune-deficiency
TAA: tumor-associated antigen
s.c.: subcutaneous injection
i.v.: intravenous injection
