# Supplementary material for: A dendritic/tumor fusion cell vaccine enhances efficacy of nanobody-based CAR-T cells against solid tumor
Source: Theranostics. 2023 Sep 18;13(14):5099–113. doi: 10.7150/thno.84946 (PMC10526666; doi:10.7150/thno.84946)
Supplement: Supplementary file 1 — Supplementary figures. [file thnov13p5099s1.pdf]

Supplementary materials

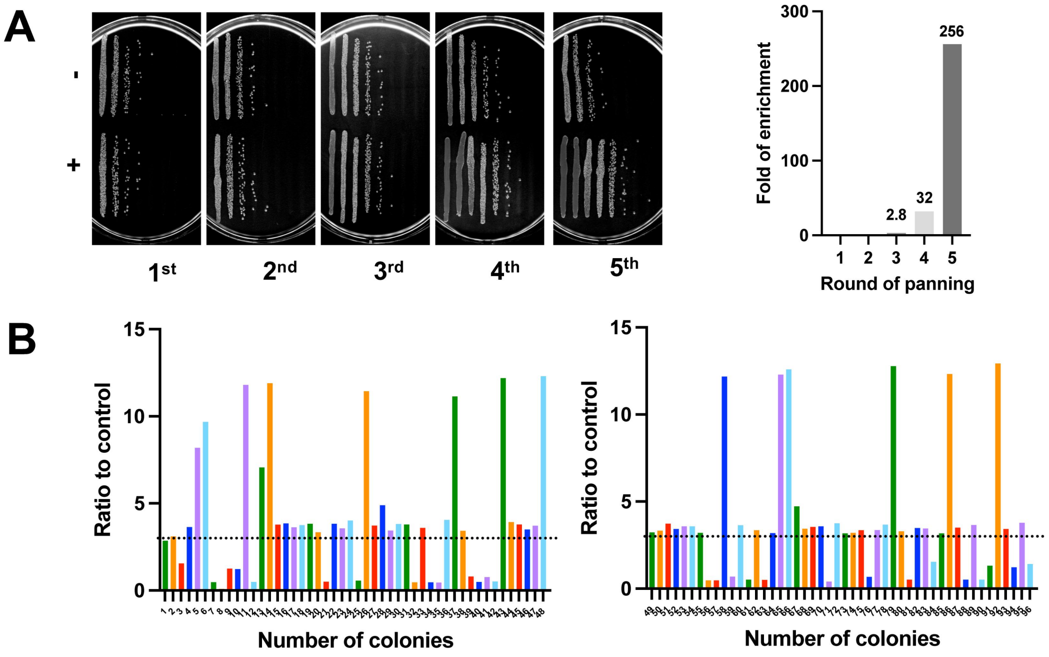

Figure S1. Screening of the VHH library for EGFRvIII. (A) The enrichment result was displayed for five rounds of panning and the statistical result for enrichment fold. +: positive screened phage; -: negative screened phage. (B) PE-ELISA for the identification of positive colonies. The ratio higher than 3 was considered positive.

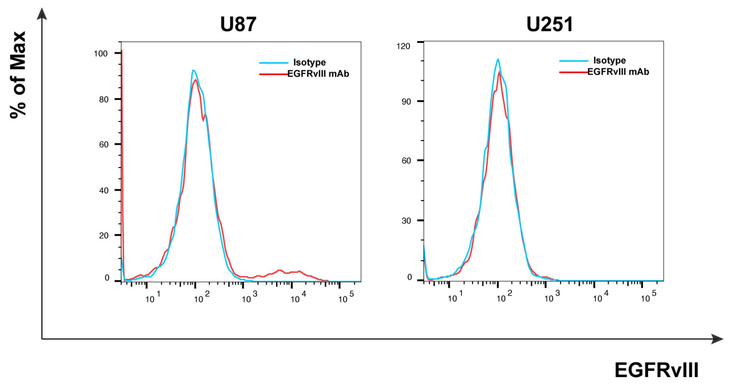

Figure S2. EGFRvIII expression of U87 and U251 was verified by flow cytometry with an anti-EGFRvIII antibody. Histogram showing EGFRvIII expression in EGFRvIII-negative GBM cells U87 and U251 cells.

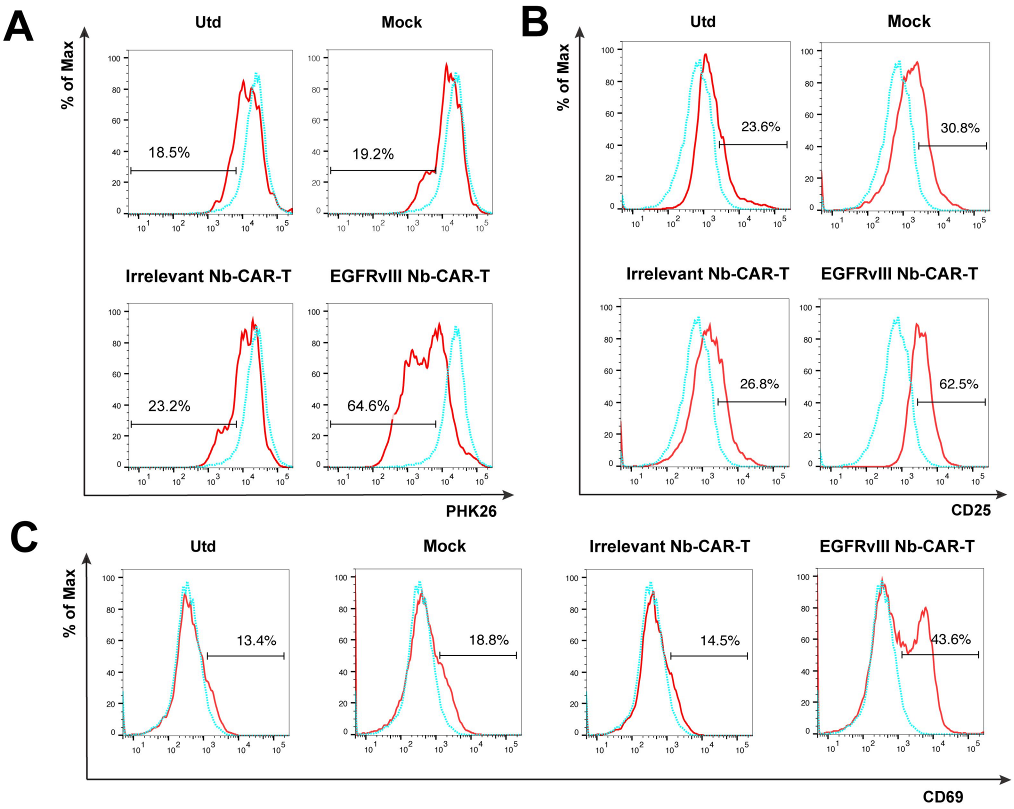

Figure S3. ***In vitro*** **proliferation and activation activity of EGFRvIII Nb-CAR-T cells.** (A) PHK26 stained EGFRvIII Nb-CAR-T cells were incubated with U251-EGFRvIII cells in culture medium for 4 days and diluted to examine their proliferation, histograms are gated on live T cells. The activation markers CD25 (B) and CD69 (C) were detected by flow cytometry analysis Representative histograms are shown.

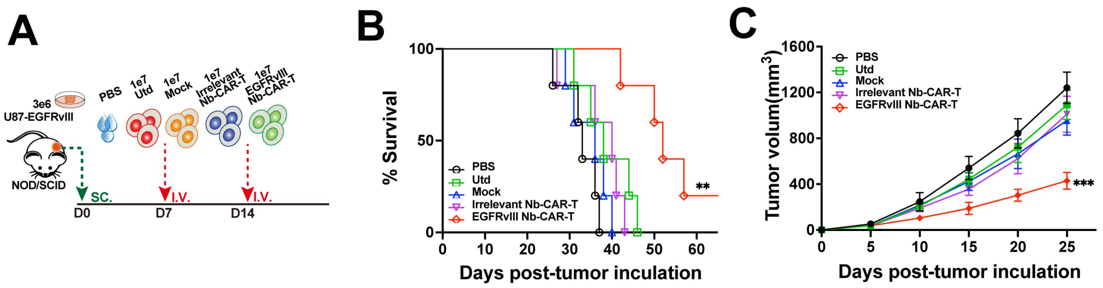

Figure S4. **Antitumor activity of EGFRvIII Nb-CAR-T cells *in vivo*.** (A) Schematic representation of the *in vivo* animal model and treatment scheme. NOD/SCID mice were inoculated subcutaneously with 3×10^6 U87-EGFRvIII tumor cells (B and C) and treated with

1×10^7 EGFRvIII Nb-CAR-T, Irrelevant Nb-CAR-T, Mock, Utd cells and 100 μL PBS as control. Tumor volume and survival ratio curve were monitored of each treatment cohort to access efficacy, n=5 per group.

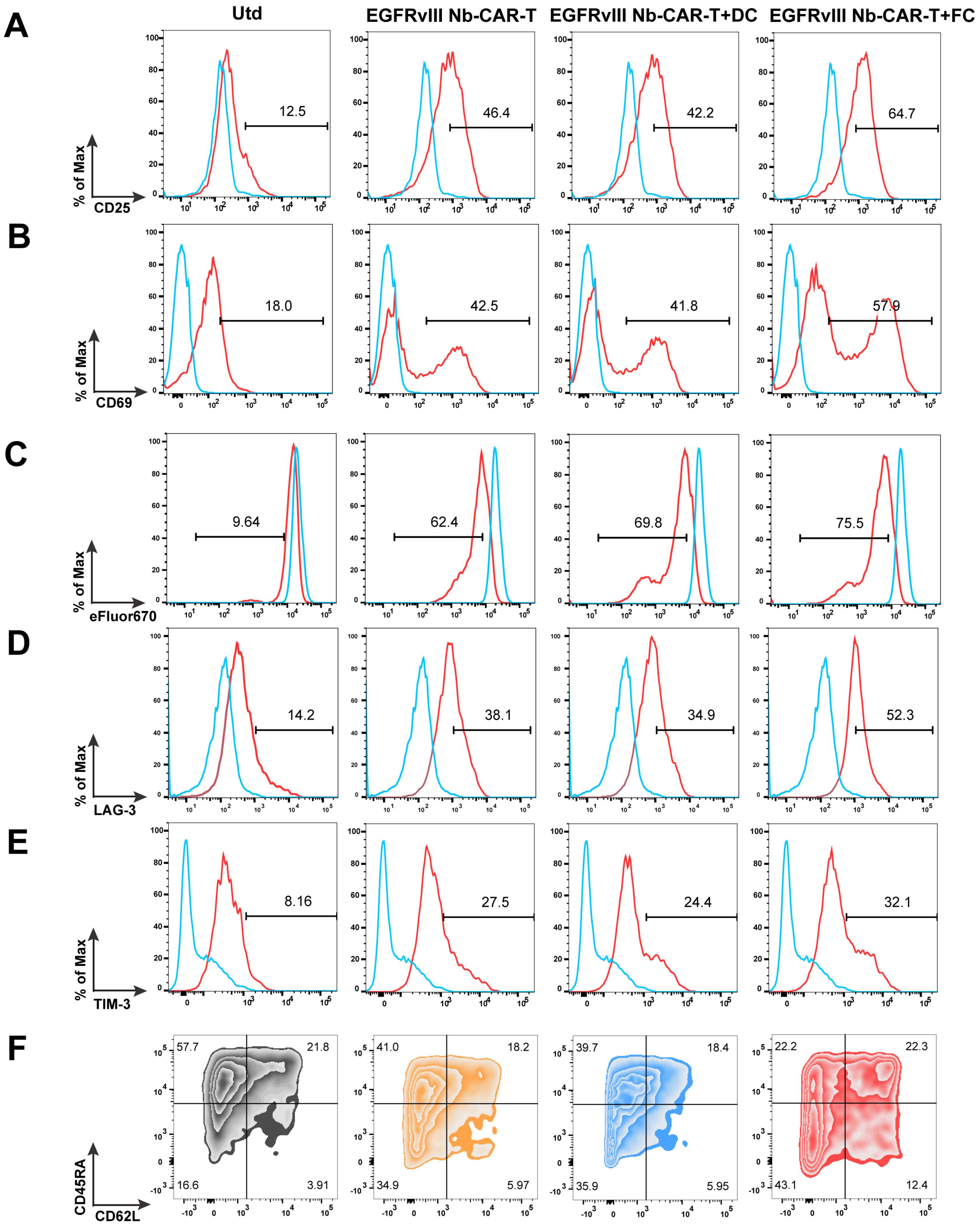

Figure S5. DC/tumor fusion vaccines can enhance EGFRvIII Nb-CAR-T effector functions *in vitro*. EGFRvIII Nb-CAR-T cells were evaluated for expression of CD25 (A), CD69 (B),

eFluor670 (C), LAG-3 (D), TIM-3 (E) and CD45RA/CD62L (F) after coculture with DC/U87-EGFRvIII fusion cells. Representative histograms are shown.

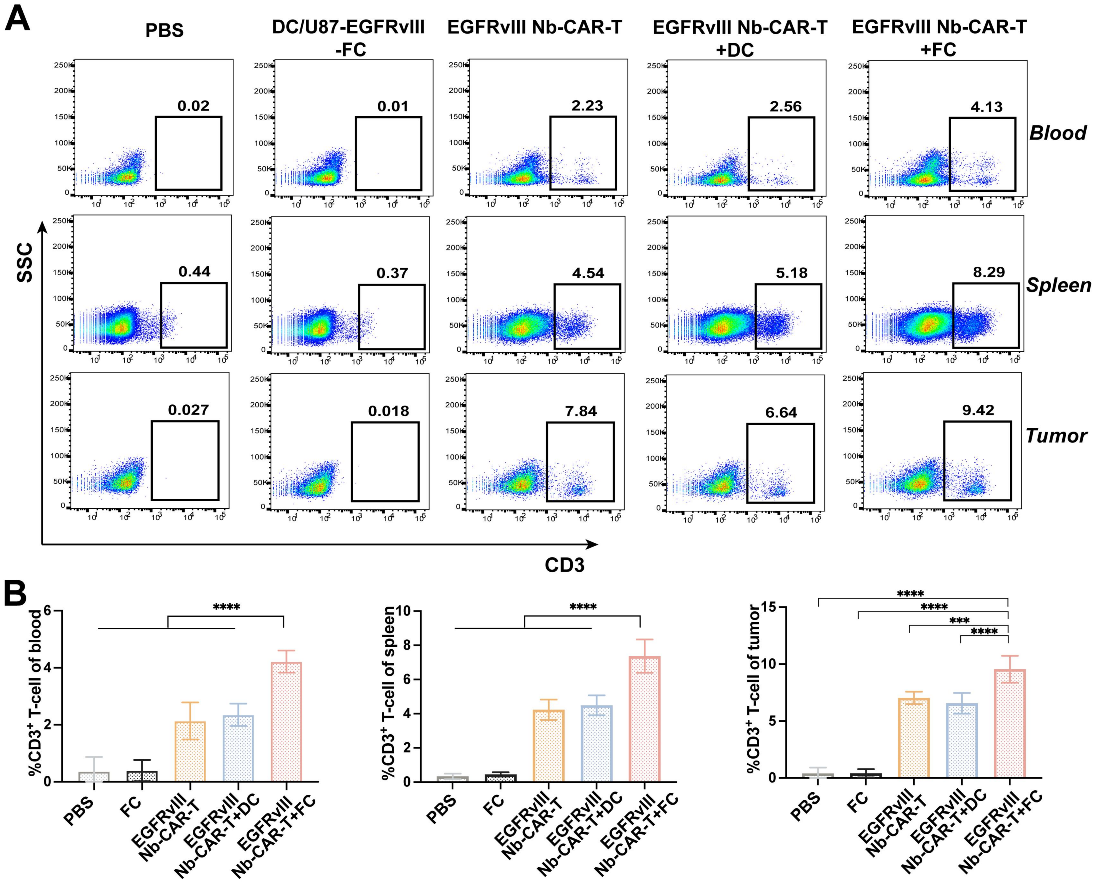

Figure S6. DC/tumor fusion vaccines enhance antitumor effects of EGFRvIII Nb-CAR-T cells therapy *in vivo*. (A and B) NOD/SCID mice were inoculated subcutaneously with 3×10^6 U87-EGFRvIII tumor cells and treated with 1×10^7 DC/U87-EGFRvIII-FC, EGFRvIII Nb-CAR-T, EGFRvIII Nb-CAR-T+DC, EGFRvIII Nb-CAR-T+FC and PBS as control groups. Representative flow plots showing the frequency and persistence of CAR-T cells in tumor issue, spleen and blood of treated mice 14 days after injection of FC and CAR-T cells, summarized results and quantitative statistics are shown. Data are presented as mean values ± SD.
